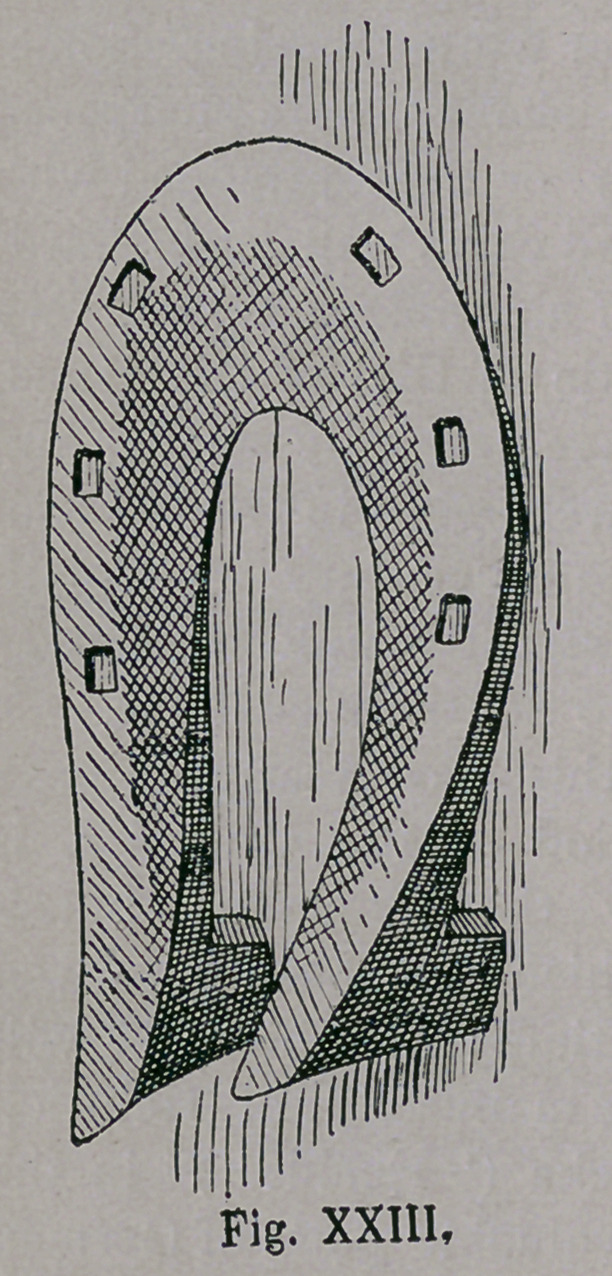# Historical Development of the Horseshoe

**Published:** 1891-08

**Authors:** 


### THE JOURNAL

OF

### COMPARATIVE MEDICINE AND VETERINARY ARCHIVES.

VOL. XII. AUGUST, 1891. NO. 8.

### HISTORICAL DEVELOPMENT OF THE HORSESHOE.

BY DISTRICT VETERINARIAN ZIPPELIUS OF WÜRTZBURG.

*Translated by S. E. Weber, V. S.**

Kind gentle steed, nobly standing,
Four shoes, will I put on your feet,
Firm and good, that you'll be fleet,
That is Donar's hammer saying.

To the woods and homeward go,
Always on the straight road thro',
Far from what is bad, still fleeing,
That is Donar's hammer saying.

Should wounds and pain become distressing,
Blood to blood shall flow,
Bone to bone shall grow,
That is Donar's hammer saying.

Carry the rider, true little steed,
Onward to all good luck bringing;
Carry him thence and back with speed,
That is Donar's hammer saying.

(*Old Meresburger Song.*)

The horse appeared comparatively late in the group of domestic animals. In searching the monuments of the ancients, which have furnished the foundation for our present culture, that is of the litoral inhabitants of the Mediterranean and of the people of Mesopotamia, we find in Egypt the first traces of the horse.

* From *Theirärztliche Mittheilungen*, organ des Vereins badischer Theirärzte, Karlsruhe, No. IV, April 1891.

But even here it appears late, on the monuments of the first ruling patricians of human origin.* Especially during the period of Memphis, (I-X Dynasty), then under the rules of Thebes (XI-XVI Dynasty), there is no trace of the horse.

It is first in the transition period, from the late rule of Thebes (XVII-XX Dynasty) to the so called period of Sut (XXI-XXX Dynasty) that there appears, in the wall pictures of the Pharaos' tombs, representations of the horse. The oldest, now known, picture of the horse is found on the walls of the tombs of Seti I. (1458-1507 B. C.) under whose reign the Israelite wandered from Egypt. The horses of these mortuary pictures are very well drawn, and have an unmistakable oriental type. There has therefore undoubtedly existed in Egypt high culture, for over 4000 years, without representation of the horse which was the next animal domesticated after the cat.

From this time on we find the horse frequently represented both by the vain-glorious Despots of Mesopotamia, and on the so called etruscan vases, which appeared after the influence of Greek art, when on almost every urn, horses in lively action and in various forms of bodily development, but almost always of an oriental type, are to be recognized. But neither here, nor in Homer, nor in the many later representations of the horse on the Roman triumphal arches etc., are to be found horses, whose hoofs have any trace of protection. Records, which describe to us the misfortunes of armies, whose horses had run their feet sore, we find on the contrary at a very early time, as in Diodorus, regarding the cavalry of Alexander the Great, in Xenophon, regarding the retreat of the ten thousand, in Polybius, regarding the cavalry of Hannibal in Etruria, etc. It is also known that the cavalry of the linguist King of Pontus, Mitaridates the Great, at times and specially at the seige of Cyzicus were delayed, in order to let the hoofs of the horses grow.

On the contrary it seems strange that of the Huns alone, whose horsemen swept over whole continents from the Asiatic highlands like a thunderstorm, such trouble had not become known either through the numerous authors of the Eastern and Western Roman Empire or from Gallia.

Horse-shoeing, very likely, was invented by different nations at about the same period during the migration of the nations, and the various kinds of new inventions were brought together in

*Until the time, Menes, with whom historical times begin, ruled in Egypt among visionary Heros or Mythological Gods.

Germany only, after each had acquired a national stamp according to climate and usefulness.

In this way come from the South, the thin, plate-like horseshoes, with staved rim, covering the whole hoof; from the Mongolian tribes of middle Asia the "Stolleneisen" (calk-shoe); whilst to our Northern ancestors, and indeed the Normans, must be ascribed, with great probability, the invention of the "Griffeneisen" (gripe-shoe) especially for the protection of the toes.

Fig. I.

All varieties of the horseshoe of southern Europe are easily distinguished from the Roman so called "Kureisen" (cure-shoe) of which several have been unearthed at various excavations and are preserved at the Romo-Germanic Museum in Mentz, (Mainz) Germany. The shoes (Figs. I. and II.) each represent thin iron plates, covering the whole hoof, which in some cases have an opening in the middle, of several centimeters in diameter.

Fig II.

These plates, apparently set forth to suit oriental and occidental body-conformation, are either directly provided with loops, or have around the outer margin a brim several centimeters high, in which rings are fastened. Through the loops or rings small ropes were drawn and in this way the shoe was fastened to the crown of the hoof and to the pastern. Sufficient securing of the toe was wanting in all these shoes and on account of this, the movement of the animal with the same, must have been very clumsy, and we can see from this that the ropes must have made the crown of the hoof and pastern sore in a short time. One of these shoes * evidently was the object of improvement, to

* Not illustrated.

prevent the animal from slipping as well as from friction, and we therefore find on it three iron cubes 1½ centimeters high, which were fastened corresponding to our toes and calks of to-day, and offer a very early ready proof, from our climatic and mountainous conditions, which later occur, principally in southern Germany, that this style of horseshoeing was not caused by error, but by a well-founded local and national interest or want.

Aside from the so called "Kureisen" (cure-shoe) for diseased hoofs we find very little from the Romans on horseshoeing or hoof-protection, and therefore we must observe special precautions with all their literature on the subject. It is because of this that I excuse Prof. Sittl's communication in the preface of Wickelmann's "Geschichte der Kunst in Alterthum" (History of Ancient Art) which contains a notice, that Fabretti in some raised work in Plazzo Matti, of a representation of a hunt by the Emperor Gallienus (Bartoli Admirand Ant. Tab. 24), showed that at that time horseshoes fastened by nails, the same as to-day, were used (Fabretti de Column. Traj. C. 7 pag. 225; Conf. Montlanc. Antiq. explic. T. 4 pag. 79). This statement proves itself erroneous, because he was not aware that the foot of the horse was repaired by an inexperienced sculptor.

How then did out of this Roman cure-shoe develop the horseshoeing of southern Europe?

Fig. III.

It was to be expected, with the Roman horseshoe, that the mode of fastening became unsatisfactory and necessitated a remedy or change. An attempt of this kind has been preserved in the so called "Asiatischen Koppeneisensole" (Asiatic Cap-iron-sole) (Fig. III.) which the Honorable Mr. Lydtin at Karlsrhue had made according to a model of the Circassian Horse Tribe Shaloks, and also according to the reverse of Lycian coins (called Triguetra).

This horse-shoe-plate likely originating in the 12th Century covers the whole surface of the sole, like the Roman shoes, with the exception of the wall region which contains a rim 1 centim.

high, and above this rises at one side towards the heel, three beak-like projections about 4 centim. high and 1 centim. wide at the base, being pointed above and turned down, which were fastened in the wall of the hoof, in the form of a hook.

This mode of fastening evidently was also insufficient and so the fastening of the shoe by nails was adopted. These iron plates used for shoes were too thin to allow nails with sunken heads to be used, so only nails with blades and cubical shaped heads were applicable. These nail-heads 6 to 8 in number, which left the toe and the back part of the heel free, served at the same time to secure the horse from slipping which the smooth plates, covering the whole hoof-surface, without doubt facilitated.

Fig. IV.

Shoes of this kind, after the old Roman style, with a very strong rim bent upwards, likely proved very comfortable for the purpose of protection, in the Sierras of the Pyrenean Peninsula, where they seem to have been in use for a long time; for in the 12th Century we find in Spain the whole form of the Roman shoe, only fastened by nails (Fig. IV. and V). At first the shoe seems to have been cut off at the heel end, but as apparently after being on for some time, bruises were noticed, the shoe was made longer at the heel, and this part was turned up so as to prevent them from becoming loose too soon, as both the Spanish horseshoes of this period show, and the acquisition was even later transferred to England (Fig. VII).

Fig. V.

The shoe containing a grove (Fig. VI.) which we shall see later, made its appearance in Germany in the 15th Century. From this time, according to our present knowledge, ceases the period of the Roman horseshoe. Its influence however, lasted a great deal longer, and has even remained until our present day.

Fig. VI.

Fig. VII.

Its successor became partly the Arabo-Turkomanic and partly the Southwest European horseshoe.

For the descendents of the Numidian light cavalry, the Roman and old Spanish horseshoe was evidently too heavy for their sandy, roadless deserts, so they made it thinner and omitted the bent up rim, because it prevented the quick movement of the horse. For the protection of the nail-heads the outer margin of the shoe was staved, so as to form a small rim on the outer surface of the shoe, thus preventing the nail-heads from being worn and the shoe lost too soon.

A horseshoe of that kind is shown by Fig. VIII. which was used in North Africa in the 12th Century, and became the model for all forms of horseshoes of the Mahometan tribes. Even now quite similar shoes (Fig. IX.) are made South and East from the Caspian Sea, at the Amu-Darja, in Samarkand, &c., which were probably introduced under Tamerlane, the conqueror of nearly the whole of Asia-Minor in the fourteenth Century.

Fig. VIII.

Fig. IX.

The so called "Samratische" (Sarmatian) horseshoe (Fig. x. and xi.) of South Russia shows in its form at the same time, traces of the last named shoe, however, greatly influenced by the Mongolian shoe, the "Goldenen Horde," which at the turn of the 16th to the 17th Century played havoc at the Volga, and the Aral. The unusual width of the toe, and especially the lightness of the iron, reminds us of the Turkomanic horseshoe, whereas on the contrary, the large bean-shaped holes, as well as the calks, were furnished through Mongolian influence.

The Sarmatian tribes were principally horsemen and it is not surprising therefore that the coat of arms of the former Kingdom of Poland in the 2nd and 3rd Quadrate shows a silver rider in armor on a silver running horse shod with golden shoes, and that at present about 1000 families in 25 lineages of the Polish Counts Jastrzembiec Bolesezy the so-called "Polnische Hufeisen Adel" (Polish Horseshoe Nobility) at the same time also carried the horseshoe on their coats of arms. The silver horseshoe in a blue field appears here as a symbol of the "Herbestpfardes" (Autumnal-horse) to which, after the christianization of Poland, was added the golden cross. The noblemen participating in the murder of the holy Stanislaus in 1084 had to carry the horseshoe reversed on their escutcheon.

From the African and Turkomanic horseshoe, through the turning up of the toes and heels, originated later, the Turkish,

Grecian and Montenegrin horseshoe of the present, as shown by Fig. XII.

Fig. X.

Fig. XI.

Fig. XII.

By the Moorish invasion in Spain the Spanish-Gothic horseshoeing was also modified, through which, the shoe became smooth, staved at the margin, very broad in the toe, and turned up at toe and heel, and at a later period the old open Spanish national horseshoe Fig. XIII. was developed; as we thus see, we can in no way deny the Arabian-Turkish origin of this shoe.

As France had received her whole culture from the South, and as the Crusades especially brought the Roman nation in close contact with them for centuries, so it cannot appear strange, that the old French horseshoe, a form of which has been preserved by Bourgelat and is represented by (Fig. XIV.), still remained in the smooth, turned up in front and behind, like the shoe of the Southern climates, with Asiatic traces, which hold on the ground, the same as all Southern shoeing, by the nail-heads.

The transit of the German Empire, in order to keep up the historical course, once more brings us back to the middle of the fifth century. At this time Attilla, the "Godegisel" (Gods' scourge), left his wooden capitol in the lowlands near the river Theis, to go to the Roman Empire and to the German and Gallican provinces, there to spread indiscribable misery to the horrors of judgment day.

The following is a prayer in those days of horror.—

> "Kleiner Huf, kleines Ross,
> Krummer Sâbel, spitz Geschoss—
> Blitzesschnell und sattlefest:
> Schrim uns Herr von Hunnenpest."

Fig. XIII.

Fig. XIV.

We are at present reminded of those times of fright, when during the clearing and tilling of the soil, a small roughly made horseshoe is found in Southern Germany, about as far as the water boundry of the Thuringian forest, and occasionally on, but principally around Augsburg, and in France as far as the Loire.

These shoes covering the margin or wall of the foot, show slight traces of having been beveled on the lower surface, and contain two bent calks very superficially placed, occasionally they are sharpened and turned in two directions. The characteristic wide bean-shaped nail-holes, are conical on the inside, and are frequently placed so near the outer margin of the shoe that from the pressure the hoofs were likely to split open. The nail-heads were shaped like a sleigh runner, and almost entirely sunk into the shoe. It evidently was not bent up at the toe, like old form of these kinds of shoes.

These shoes, according to our conception of to-day, were so carelessly finished that in the scientific circles of historical researches they were, until very recently looked upon as saddle mountings or something similar and not as horseshoes.

This shoe was for some time, while it was plentifully found in

France, regarded as of Celtic make; but this is certainly not the case, as it is of Hunish and Hungarian "Nationalitat" (Nationality). An exactly scientific proof, it is true, according to our present knowledge, cannot be furnished; however, it will stand well enough until the error is proven.

Fig. XV.

This peculiar kind of horseshoe, has been found in South Germany and North-east France, as far as the region of Orleans, where, as it has been proven the Huns appeared. This therefore, speaks for their descendants: 1st, The far extended and yet sharply limited places of finding the shoe; 2nd, The small size, corresponds to the historically proven smallness of the Hunisch horse; 3rd, The hasty and careless make, which does not indicate that it was made by settled workmen; 4th, The horseshoe (Fig. xv.) be-speaks the Hunish workmanship of the present Chinese shoe, which, in making of the nail-holes, shows to-day, related touches of the productions of the Mongolian Ancestors.

Aside from the peculiar shaped nail-holes, the characteristic of the Hunish shoe, consists in the changes of the calks for summer and winter shoeing, as well as in the sinking of the nail-heads. The Huns therefore, aside from the indistinctly marked attempts of the Romans in this direction which are the only ones known to me, must be regarded as the inventors not only of the calks, (but partly next to the Normans) also of the sharpened winter shoeing, and of the not unimportant invention of sinking the nail-heads observed in Fig. xv.

Fig. XVI.

The Hunish shoeing was therefore an important invention for the Germans. After centuries later, wherever horse-shoeing was practised, it was done solely according to Hunish methods ; where by the shoe was very possibly made heavier, was more carefully finished and in course of time showed an attempt to bend the toe (Fig. XVI. a).

Fig. XVI a.

In the Bomberg Dom we find an equestrian statue, not unknown in the history of art, which was formerly held to be that of Emperor Conrad III ; at present however, the opinion prevails generally that it represents "Stephen I, den Heiligen" ( Stephen I, the Saint.)

Stephen I, the first King of Hungary formerly was a heathen and was named "Najk". He reigned from 997 to 1038, his important events were the many victorious wars led against rebellious chieftains of his country, and he was canonized in 1087. His equestrian monument in Bomberg Dom was, in consequence hardly made before the year 1087. Notwithstanding that the Huns had been defeated 500 years before on the plains of Catalania, the horse of the above mentioned monument carries, as I have convinced myself personally, Hunish horseshoes, modified however, by blade shaped calks just then coming into use. This is proof that, at least in Hungary the Hunish method of shoeing, was preserved an extraordinary long time. By this it has not become improbable, that at least the many shoes of this kind, which were found on the Lechfield, come not directly from the Huns, but from their successors, the Hungarians, whose invasions took place in the first half of the 10th Century.

About the same time of the Hungarian invasions, the Normans began to disturb the South-western part of Europe with their Viking expeditions. Their Sea-King's seem to have been equestrians at very early times, and to have had their horses shod, although perhaps only in winter; at least the excavation of the Viking ship in 1881, disclosed the remains of a horse which was shod. The shoeing consisted only of a toe protection—"Brodder" ( Bruder )—( Brother )—provided with a small sharp calk, and fastened by two nails.

When later in the year 1130, the Norwegian king Sigard Yorsalafar, during his journey to Jerusalem, entered Constantinople, his horse is said to have carried only the small toe-protecting shoes.

The art of horseshoeing, immediately after the migration of the nations, came near our improvement of the same to-day; especially near the reputed discoveries met with, which consist simply of iron protection for the margin of the hoof, fastened by nails. The heads were sunk into the shoe so as to increase its firmness. Special consideration was given to local and climatic conditions through the introduction of toes and heels.

Fig. XVIII.

The mechanism of the hoof also found remarkable consideration, inasmuch that they apparently avoided driving nails too close to the heel end of the shoe. Notwithstanding this early improvement in the art of horseshoeing, the Huns (as stated before) took a prominent part. It appears to have taken a long time after the migration of the nations for shoeing to become general, as is shown by various descriptions of tournaments, picture of horses, etc.

Fig. XIX.

We will mention in the first place the "Percival des Wolfram Von Eshenbach," as well as "Christ. Von Troies," where there is a great deal said about horses, horse-grooms, and tournaments, but nowhere in those works is any mention made of horseshoeing. Likewise is found the horse on the coat of arms, of Wolfram Von Eschenbach, in the Manessi collection in Paris; which was begun in Switzerland in the 14th century; but, although, we find this horse most beautifully finished, it was not shod.

During the time of the Crusades, 1096-1291 ; however, there appeared suddenly in Germany, a plate-like horseshoe of Southern character (Figs. XVIII. and XIX.), which was occasionally bent upward at the heel end, and was very heavy. The toe was very broad sometimes, and was also bent upwards. In this form we have seen the shoes of the Balkan and Pyrean Peninsula. The shoe was remarkably narrow at the heel, and was supplied with calks. which accounts for the highness of the back part of the shoe, Frequently we find one calk set diagonally, but the other drawn out wedge shaped, and sharp ; so that there existed a great similiarity between this iron shank and that used by Count Einsiedel for winter shoeing. Sometimes both shanks were sharpened in this way, or were provided with blade-shaped calks well set forward. The form of nail-holes used was very characteristic of that of the Huns, but they were decidedly smaller and square, as were seen in the African shoe of the 12th century. The nail-heads were slightly sunk, which was according to Southern customs.

Fig. XX.

Fig. XXI.

That this shoe really belongs to the period of the Crusades, is proven by the numerous horse-pictures which have been preserved from that time ; of which we will mention the manuscript of Heinrich Von Veldecka ("Eneidt")* in the year 1180, which belongs to the most valuable parts of German history of art.

This South European-Hunish horsehoe, had remained the standard form during the middle ages and until the 30 years

* "Wanderungen des Aeneas" (Travels of Aeneas).

war, at least in South Germany. The shoe was continually improved, and reached its highest point of perfection, about the time of the "Bauernkrieg" (Revolution of the Peasants) at a time when, under the leadership of the Renaissance, the whole art of mechanics, and especially that of blacksmithing had taken an extraordinary great stride (Figs. xx. and xxi.).

Fig. XXII.

Fig. XXIII.

The shoe (Figs. xxii. and xxiii.), is found in Franconia, in all places, where in the 16th century, battles had been fought with the rebellious peasants. We may, therefore, be justified in fixing its origin mainly, from that period, for which also speaks its high perfection of form. We find here still, the bent up heel and toe, (the latter broad and thin) of the south European form.

The staved rim of the Spanish-Arabic-Turkomanic shoe, is observed to be undergoing a change to that of a groove. The broad surface of the shoe evidently led to the bevelling of the same, so as to lessen sole pressure. The size of the nail-holes remains still like that of the Huns; but the unsunk Southern nail-heads, yet serve to improve the hold on the ground. The calks were next placed forward, perhaps from an uncultivated sense of beauty, or from the high bending up of the hind part of the shoe, which would necessitate a high and heavy unsightly calk.

From this time on horseshoeing in south Germany fell back very quickly, and loses all scientific holds of support after the 30 years war. In the meantime, toe protection in the form of a calk had spread from the colder north over southern Germany; whereas this north-German invention did not find favor in England in consequence of her mild oceanic climate. Also, the calks in England, as well as in the southern countries, on the same ground, therefore with good reason, could at no time be adopted. This did, however, not interfere with the use of the calk in the colder south-Germany, where after a use of nearly 1500 years it has maintained its local and climatic adaptation. Notwithstanding the occasional apeing by foreigners, it has remained victorious in its original form, and has been chosen in many countries.

The historical development of the horseshoe in general, from about the time of Emperor Maximilian until the seven years war, furnishes a true picture of the confused condition of things at that period of time, which to make intelligible, would require a separate and complete treatise. Interesting as it is to the scientist, to follow up this development, and mode of present German horseshoeing, which aside from the National toe and calk, is the English form and has become influential and with full right, for a periodical of this kind further, more comprehensive statement, would under all circumstances take up too much room ; wherefore, I must drop the pen, although reluctantly.

## Figures and Tables

**Fig. I. f1:**
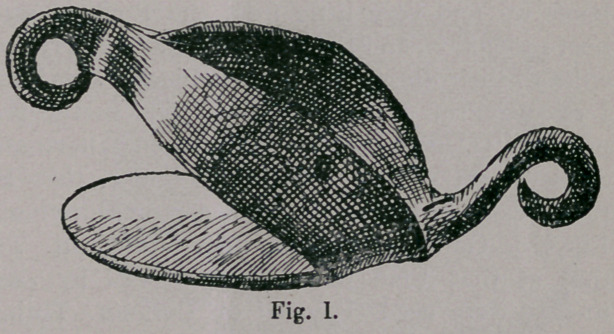


**Fig. II. f2:**
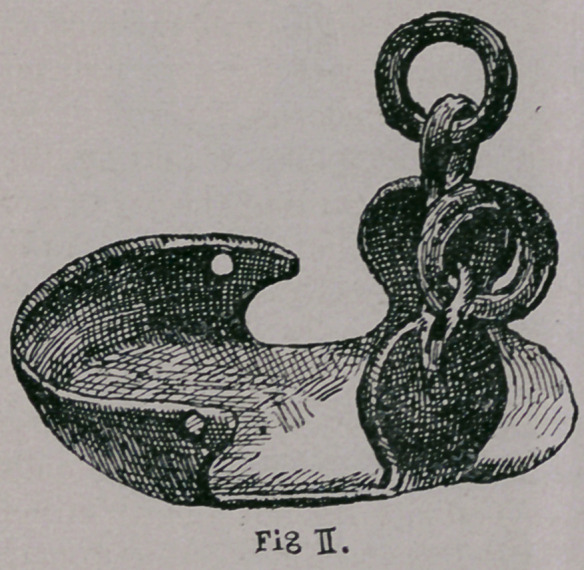


**Fig. III. f3:**
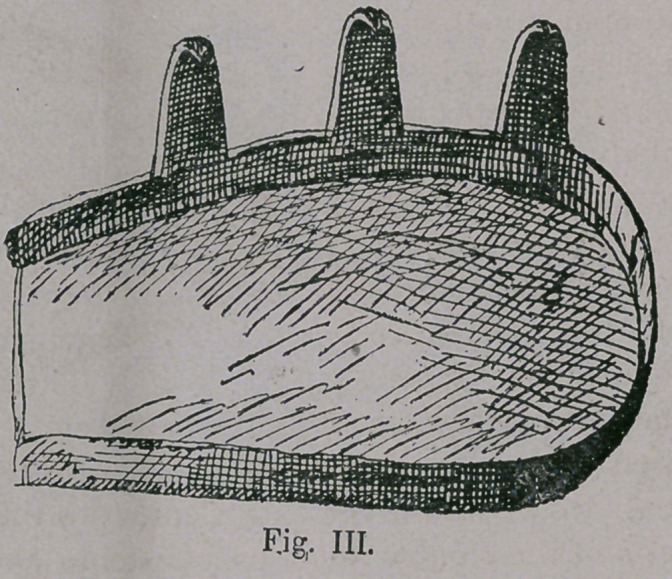


**Fig. IV. f4:**
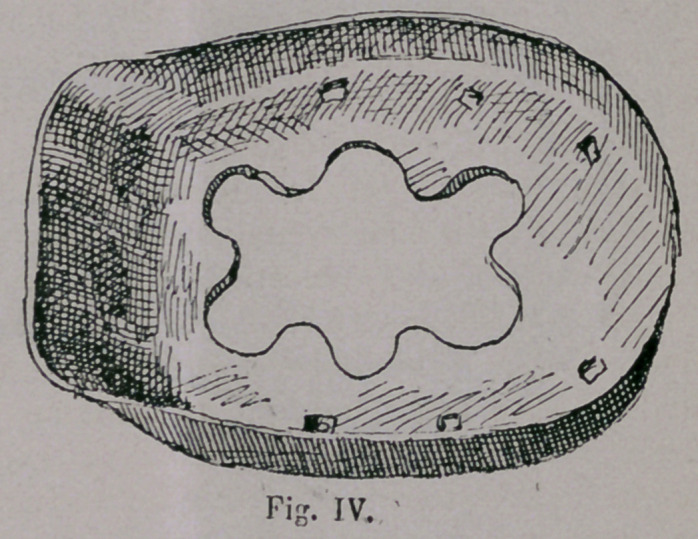


**Fig. V. f5:**
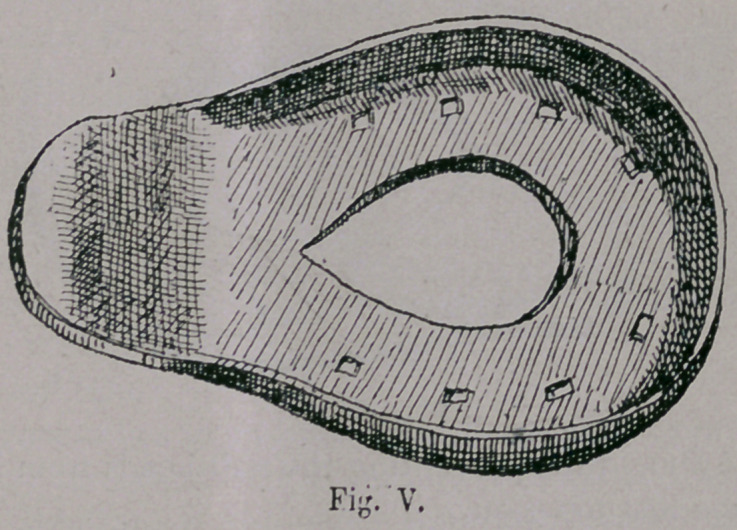


**Fig. VI. f6:**
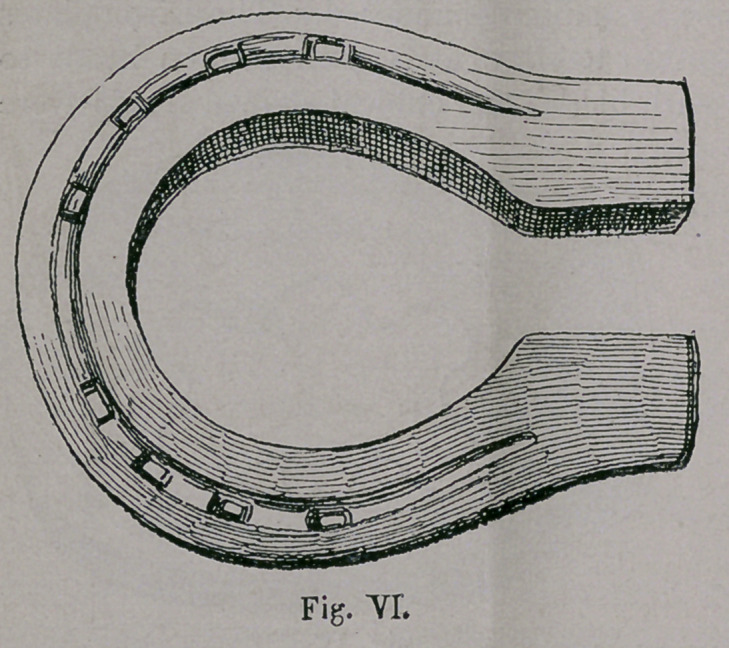


**Fig. VII. f7:**
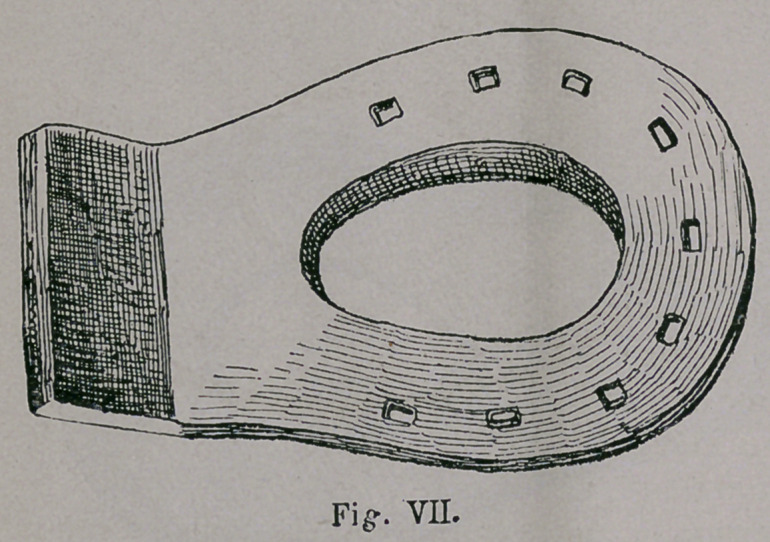


**Fig. VIII. f8:**
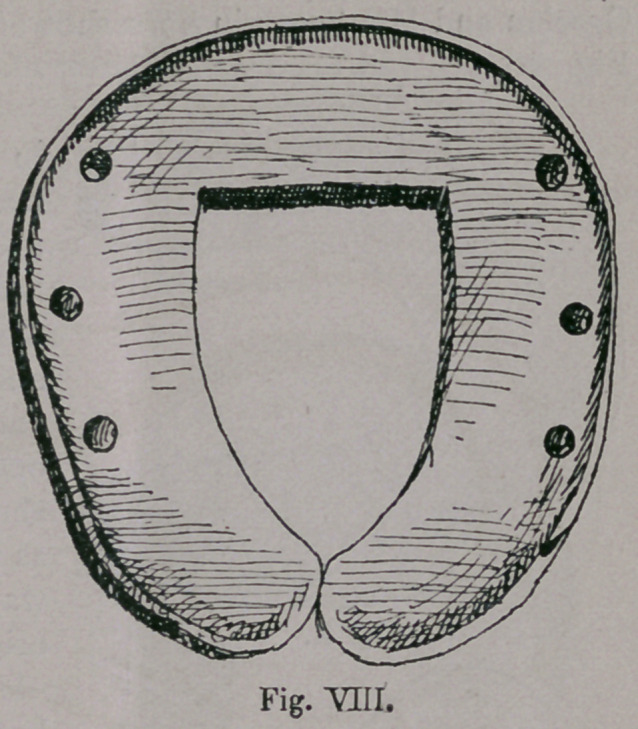


**Fig. IX. f9:**
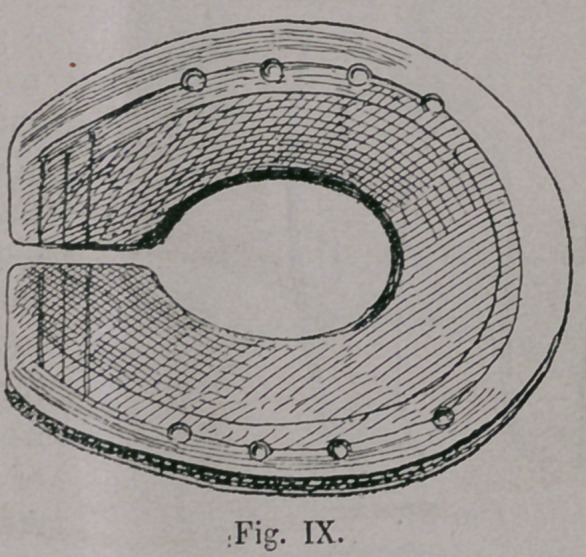


**Fig. X. f10:**
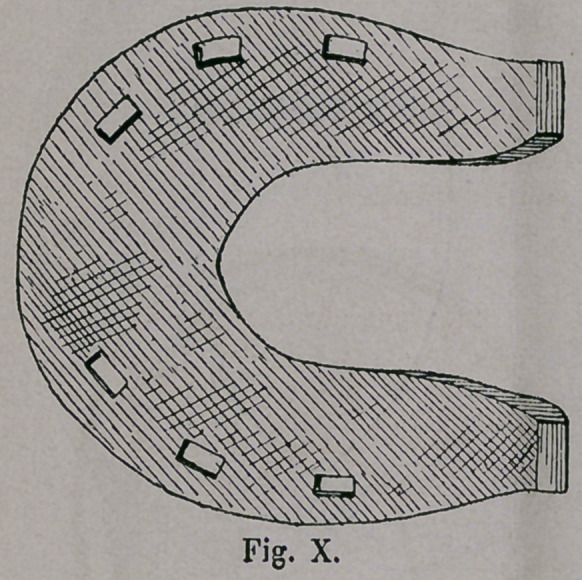


**Fig. XI. f11:**
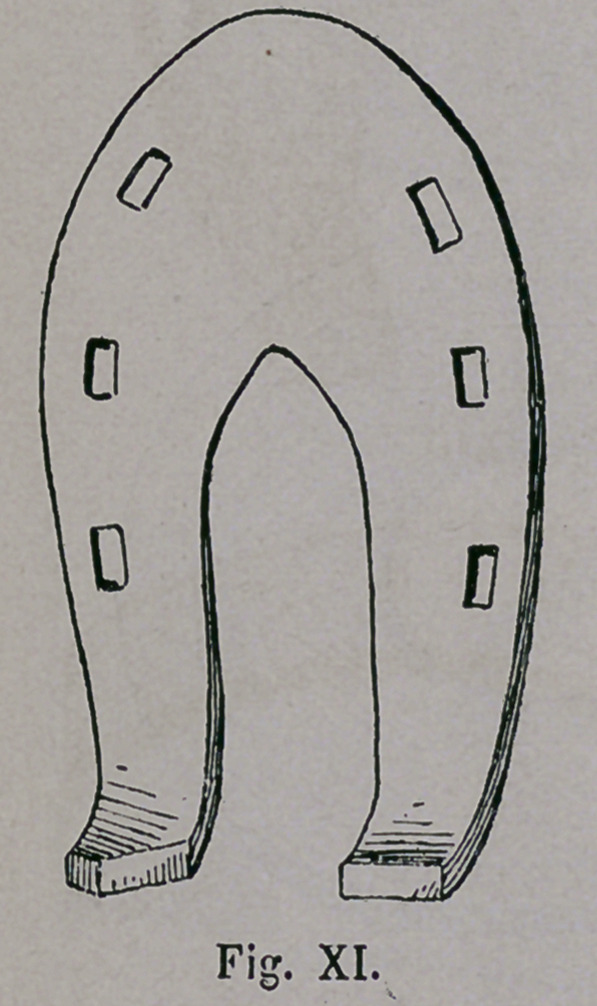


**Fig. XII. f12:**
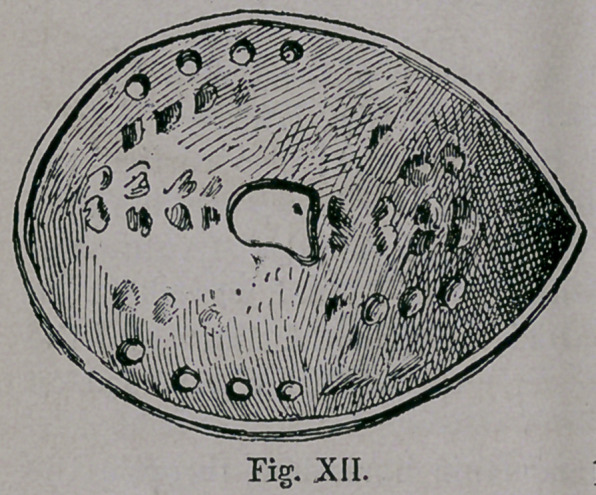


**Fig. XIII. f13:**
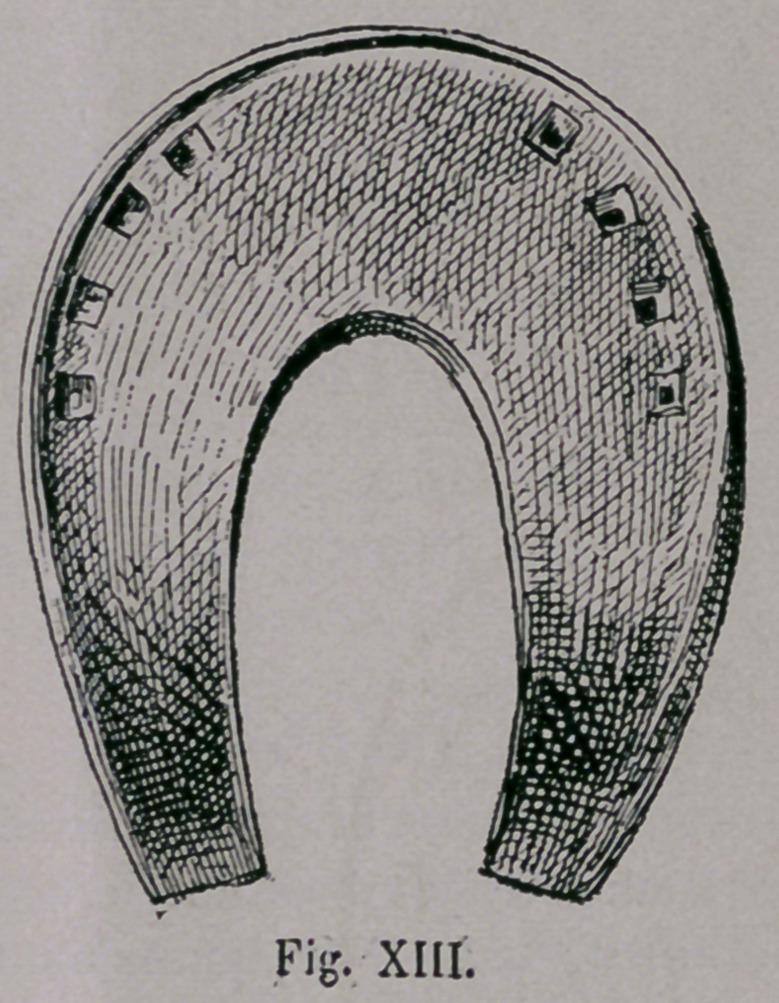


**Fig. XIV. f14:**
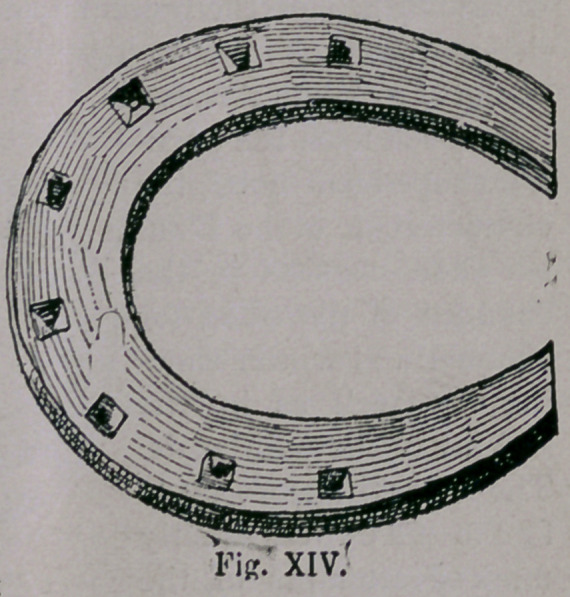


**Fig. XV. f15:**
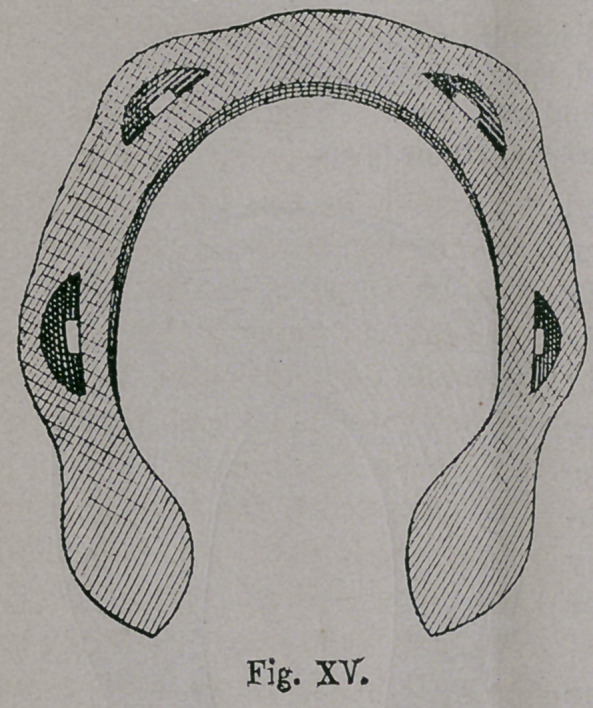


**Fig. XVI. f16:**
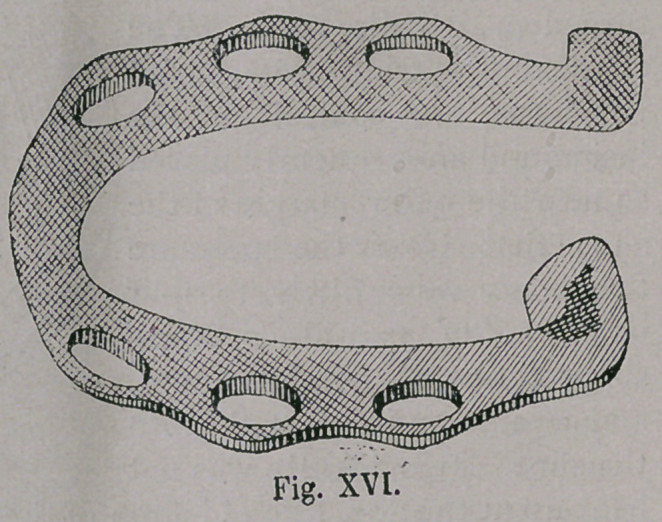


**Fig. XVIa. f17:**
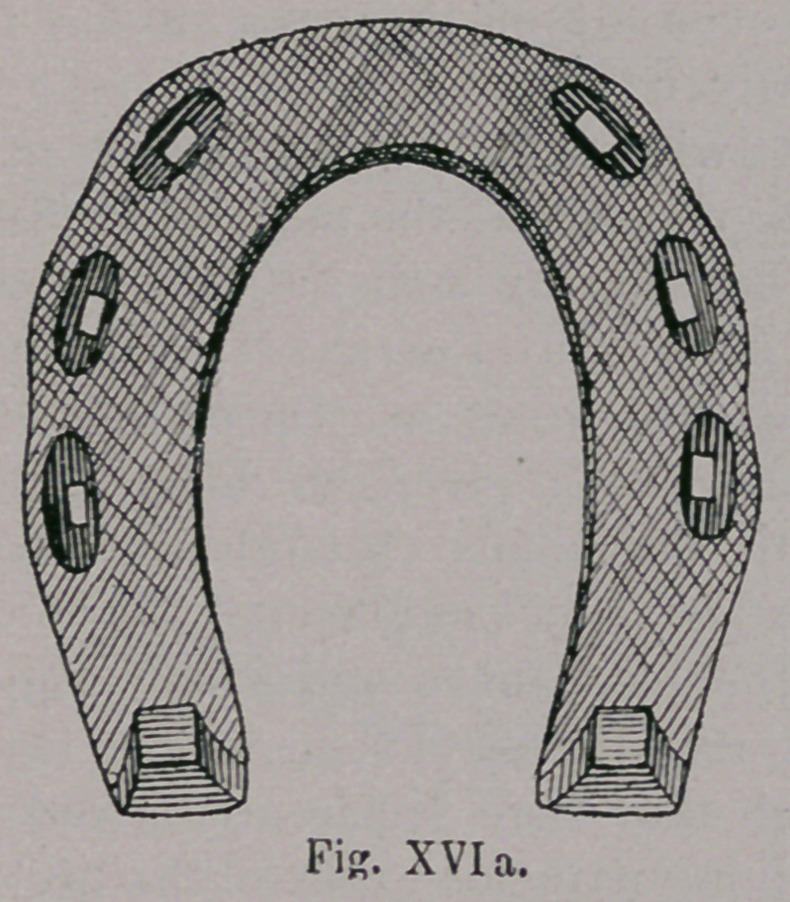


**Fig. XVIII. f18:**
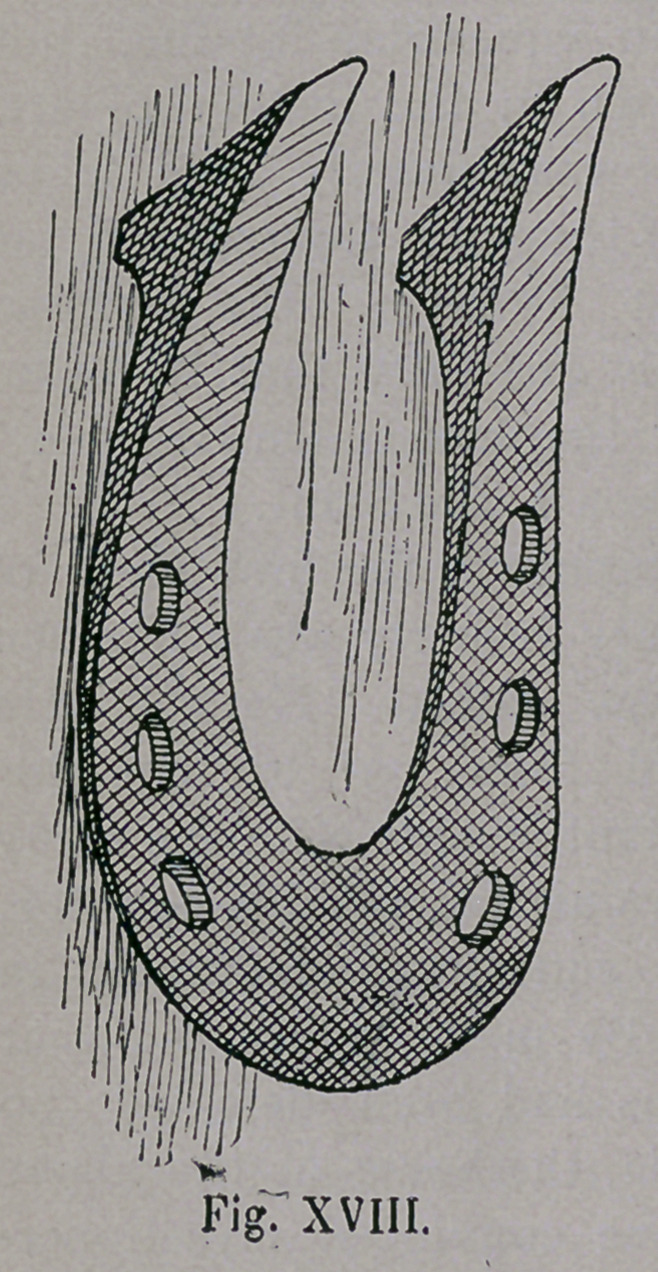


**Fig. XIX. f19:**
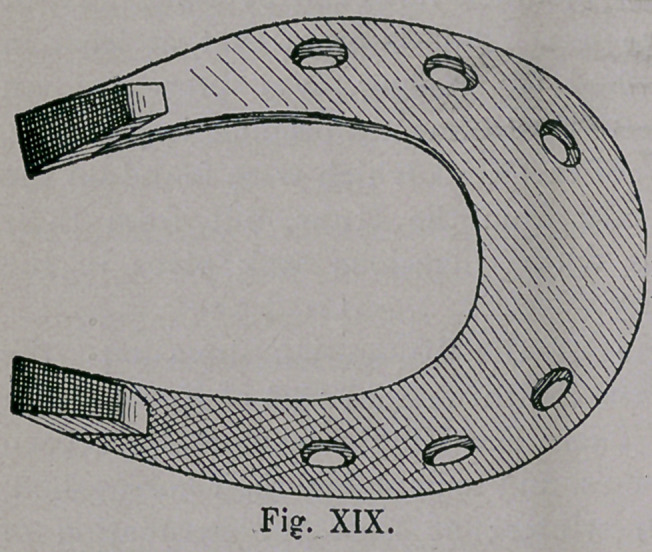


**Fig. XX. f20:**
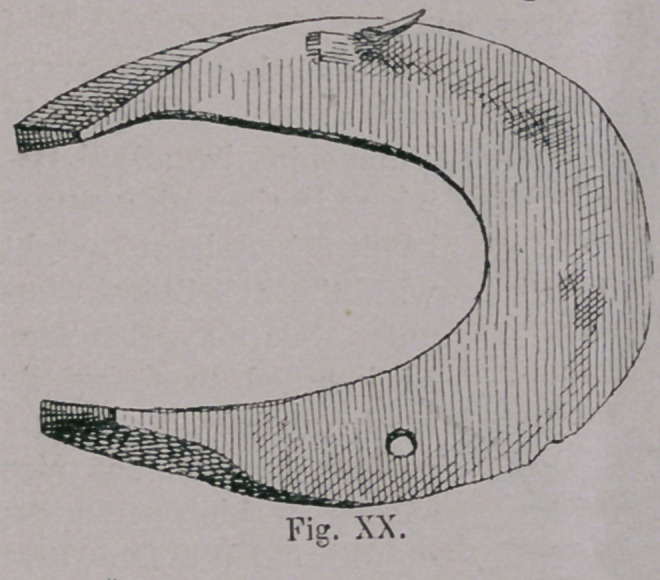


**Fig. XXI. f21:**
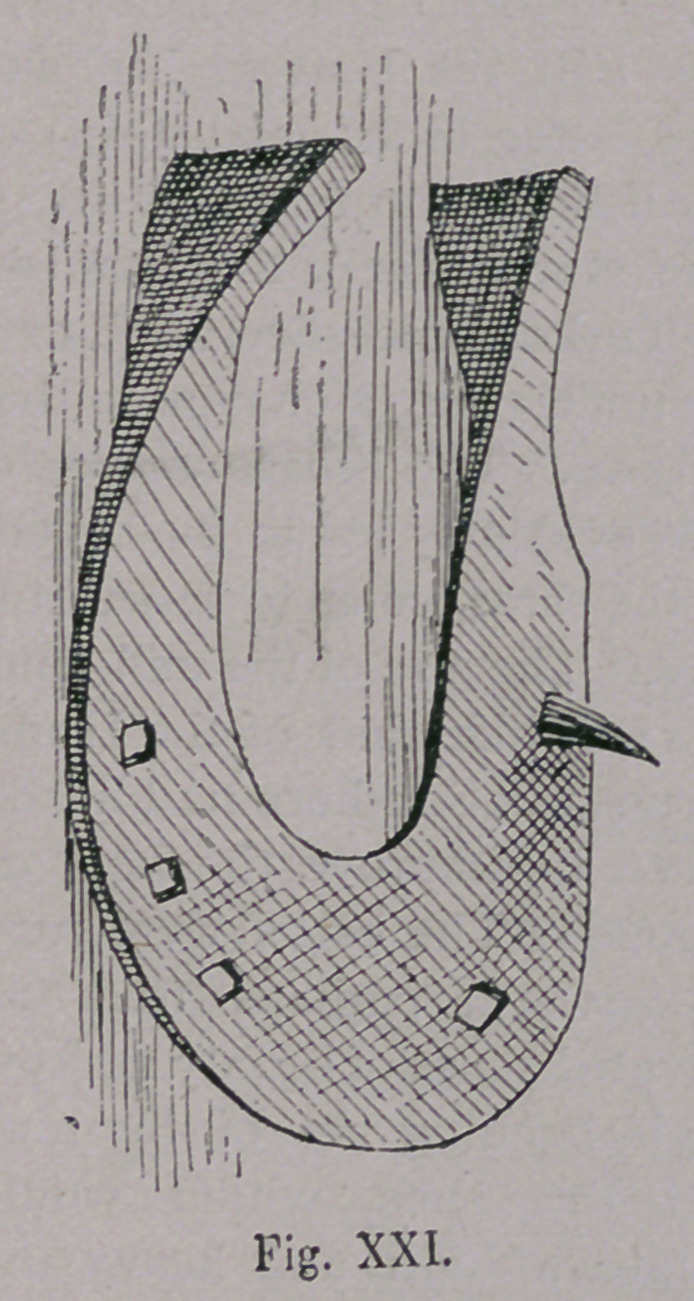


**Fig. XXII. f22:**
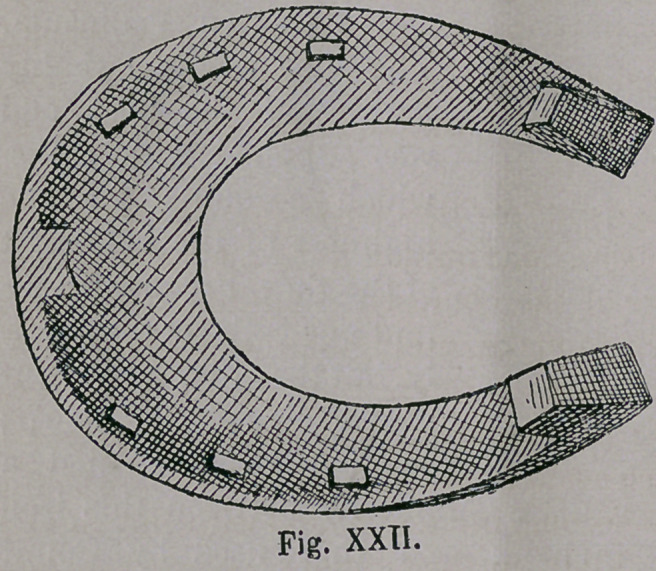


**Fig. XXIII. f23:**